# Human umbilical cord mesenchymal stem cells deliver exogenous miR-26a-5p via exosomes to inhibit nucleus pulposus cell pyroptosis through METTL14/NLRP3

**DOI:** 10.1186/s10020-021-00355-7

**Published:** 2021-08-19

**Authors:** Xiaoqiu Yuan, Tiefeng Li, Lei Shi, Jinhao Miao, Yongfei Guo, Yu Chen

**Affiliations:** Spine Center, Department of Orthopaedics, Changzheng Hospital, Naval Medical University, No 415 Fengyang Road, Shanghai, 200003 China

**Keywords:** Intervertebral disc degeneration, N^6^-methyladenosine, Umbilical cord mesenchymal stem cells, Exosomal miR-26a-5p, Pyroptosis

## Abstract

**Background:**

Intervertebral disc degeneration (IVDD) is the breakdown of the discs supporting the vertebrae. It is one of the most frequent causes of back pain worldwide. Currently, the clinical interventions for IVDD are mainly focused on symptom releases. Thus, new therapeutic options are needed.

**Methods:**

Nucleus pulposus (NP) samples were obtained from 20 patients experiencing IVDD and 10 healthy volunteers compared for mRNA N^6^-methyladenosine (m^6^A) mRNA modification as well as methyltransferase (METT) like METTL3, METTL14, and Wilms’ tumor 1-associated protein mRNA and protein abundance following exosomes exposure from mesenchymal stem cells. In addition, microRNA expressions were also compared. The correlation between the NLR family pyrin domain containing 3 (NLRP3) and METTL14 was measured by luciferase reporter assay. Cytokines were evaluated using an enzyme-linked immunosorbent assay. METTL14, NLRP3, and insulin-like growth factor 2 mRNA-binding protein 2 mRNAs were measured via a quantitative reverse transcription-polymerase chain reaction. Protein was assayed using western blots. Cell death was assessed by propidium iodide staining, lactate dehydrogenase release, gasdermin-N domain abundance, and caspase-1 activation.

**Results:**

The human umbilical cord mesenchymal stem cell (hucMSC) exosomes were found to effectively improve the viability of NP cells and protect them from pyroptosis through targeting METTL14, with a methyltransferase catalyzing m^6^A modification. METTL14 was highly present in NP cells from IVDD patients, which stabilize NLRP3 mRNA in an IGFBP2-dependent manner. The elevated NLRP3 levels result in the increase of interleukin 1β (IL-1β) and IL-18 levels and trigger pyroptotic NP cell death. Such pathogenic axis could be blocked by hucMSC exosomes, which directly degrade METTL14 through exosomal miR-26a-5p.

**Conclusions:**

The results of the current study revealed the beneficial effects of hucMSC exosomes on NP cells and determined a potential mechanism inducing IVDD.

**Supplementary Information:**

The online version contains supplementary material available at 10.1186/s10020-021-00355-7.

## Introduction

Intervertebral disc degeneration (IVDD) is the breakdown of the pads (*discs*) between vertebrae, which provide cushion and mechanical support for the vertebrae. The disc itself is avascular and consists of a gel-like center called the nucleus pulpous (NP), which is surrounded by a tough, fibrous cartilage ring called the annulus fibrosis (Colombier et al. 2014). They are a common site for injury and degradation despite the robust structure of the intervertebral discs. N6-methyladenosine (m^6^A) is the most common endogenous RNA modification in mammalian cells and can efficiently regulate RNA abundance and gene expression levels by modulating a variety of aspects including RNA secondary structure and metabolisms (Huang et al. 2018). It is mediated by a methyltransferase complex or *writers*, including methyltransferase (METTL) like METTL3, METTL14, and Wilms’ tumor 1-associated protein (WTAP) (Shen et al. 2020). In addition, m6A can be demethylated by the *erasers* such as FTO and ALKBH5 (Fang et al. 2021). Furthermore, *readers* such as the YTH family proteins and insulin-like growth factor 2 mRNA-binding proteins (IGF2BP) including IGF2BP1-3 recognize and bind m6A sites, leading to different destinies of target RNA (Shen et al. 2020; Shi et al. 2021b). However, little is known about the role of m6A modification in IVDD development.

The strongest biological link to IVDD is inflammation, which results primarily from alterations in the cytokine milieu surrounding the intervertebral disc such as interleukin 1β (IL-1β) and IL-18 (Risbud and Shapiro 2014; Johnson et al. 2015). Nucleotide-binding oligomerization domain-like receptor family protein 3 (NLRP3) is a cytoplasmic protein that functions primarily as a sensor for pathogens (Martinon and Tschopp 2005) and also responds to metabolic stress and tissue damage (Leemans et al. 2011). NLRP3 inflammasome is a multiprotein complex and mediates inflammatory responses by activating caspase-1 for processing premature IL-1β and IL-18 (Shi et al. 2021a). NLRP3 recruits and activates caspase‐1 after the identification of pathogen molecular pattern, cleaves gasdermin D (GSDMD) to form GSDMD‐N, and induces cell membrane perforation, cell rupture, and release of contents, causing pyroptosis (Wang et al. 2020b). Indeed, NLRP3-mediated pyroptosis occurs in intervertebral discs themselves (He et al. 2020).

Exosomes are small extracellular vesicles that contain constituents of origin cells (Kalluri and LeBleu 2020) and can be selectively taken up by cells and therefore transfer cargos to reprogram the recipient cells upon their bioactive compounds (Zhang et al. 2019). Many studies have investigated the function of exosomes from different sources including mesenchymal stem cells (MSCs-exosomes) and human umbilical cord MSCs (hucMSCs-exosomes) (Mianehsaz et al. 2019). To date, a potential treatment option is using exosomes extracted from human MSCs because they function through microRNA (Zhang et al. 2020). Among the multitude of potentially relevant microRNA functions, a microRNA panel has been identified to accelerate vaginal epithelial cell growth and reduce apoptosis, suggesting a possible avenue for tissue restoration or a reduction in cell loss as found in the NP (Zhu et al. 2019). miR-26a-5p serves as an oncogenic miRNA in different cancer cells by inhibiting its targets to promote cell proliferation and inhibit cell death (Ye et al. 2020). miR-26a-5p also inhibits the progression of atherosclerosis (Ren et al. 2020); improves autophagy, inflammation, and oxidative stress of cerebral ischemia–reperfusion injury (Yang et al. 2020); and inhibits cell apoptosis and inflammatory responses in lipopolysaccharide-induced acute lung injury (Li et al. 2021; Wang et al. 2021). In particular, serum miR-26a-5p is upregulated in injury‐induced disc degeneration animal models and modulates Smad signaling in vertebral discs (Fan et al. 2019).

Given the number of potential factors in IVDD development are rarely described beyond correlation, the current study sought to elucidate a complete pathway that includes several potentially targetable elements. Here, the role of miR-26a-5p, which was discovered via screening microRNA secreted by human umbilical cord stem cells on an mRNA-regulated inflammasome cascade that modulates pyroptosis in IVDD, was examined.

## Materials and methods

### Clinical sample collection

The study protocol received approval from the Ethics Committee of Changzheng Hospital, Naval Medical University. Written, informed consent was obtained from all study participants. Degenerative NP samples were collected from 20 patients with IVDD, who underwent surgical treatments at the Changzheng Hospital, Naval Medical University from March 2015 to April 2018 (Additional file 1: Table S1). Normal NP samples were collected as controls from 10 non-IVDD patients, who underwent idiopathic scoliosis or anterior decompression surgery due to fresh traumatic lumbar fractures with neurological deficits. The NP tissues were placed into a cell culture medium followed by a wash with phosphate-buffered saline before quantitative reverse transcription-polymerase chain reaction (qRT-PCR) and Western blot analysis.

### Cell culture

Passage 2 generation of hucMSC was received from Cyagen Biotechnology Inc. (Item: HUXUB-01001 lot number: 130606L01, Cyagen Biotechnology Inc., Guangzhou, Guangdong, China). Cells were maintained in MSC NutriStem® XF Medium (Biological Industries, Beit HaEmek, Israel) containing 15% fetal bovine serum (FBS; Gibco, Grand Island, NY, USA) at 37℃ under 5% CO_2_ in the air, replacing the culture medium every 2–3 days. HucMSC at passage 3 was used for subsequent experiments. The immunophenotype of the culture‐expanded hucMSC was characterized using flow cytometry based on previously described methods (Liu et al. 2012). All hucMSC showed positive expression for CD29, CD44, CD73, CD90, and CD105 and negative expression for CD11b, CD14, CD34, CD45, and HLA-DR. The adipogenic and osteogenic differentiation of hucMSC was assessed by Oil red O staining and Alizarin Red staining, respectively (Yuan et al. 2020). Human NP cells (HNPC) were isolated and cultured as previously reported (Dudek et al. 2017). After washing thrice with sterile phosphate-buffered saline (PBS), annulus fibrosus was meticulously eliminated from the human intervertebral disc tissues. Cells were cultured with media replacement every 72 h and passaged at 80%–90% confluence. HNPC at generation 2 or 3 was used herein.

### Cell transfection

pLKO.1 lentiviral vectors containing shRNA targeting human METTL14 were synthesized by Sangon Biotech (Shanghai, China). The sequences of shRNA targeting METTL14 are found in Table 1. METTL14 or NLRP3 ectopic expression vector was produced by inserting the coding sequence of METTL14 or NLRP3 into pLVX-Puro. A total of 293 T cells were seeded into a six-well plate and were transfected with pLKO.1-METTL14-shRNA (shMETTL14), pLVX-Puro-METTL14 (METTL14), or pLVX-Puro-NLRP3 (NLRP3) for 4–6 h using lipofectamine following kit instructions. Lentiviral particles were harvested 48 h after transfection and were employed for transduction into HNPC. pLKO.1-scramble shRNA and blank pLVX-Puro (vector) were used as negative controls.

**Table 1:** shRNA and siRNA sequences used in this study

shRNA/siRNA	Sequences (5′-3′)
shMETTL14-1	GCATTGGTGCCGTGTTAAA
shMETTL14-2	GGATGAACTAGAAATGCAA
siIGF2BP1-1	GGACUUGGAGAAAGUGUUUTT
siIGF2BP1-2	GGCUCAGUAUGGUACAGUATT
siIGF2BP2-1	CCCAGUUUGUUGGUGCCAUTT
siIGF2BP2-2	GCGAAAGGAUGGUCAUCAUTT
siIGF2BP3-1	CCUUGAAAGUAGCCUAUAUTT
siIGF2BP3-2	GCUGCUGAGAAGUCGAUUATT

HNPC were transfected with siRNA targeting IGF2BP1, IGF2BP2, or IGF2BP3 (Sangon Biotech) using Lipofectamine 2000 (Invitrogen, Waltham, MA, USA) according to the manufacturer’s protocol. The sequences of siRNA targeting IGF2BP1, IGF2BP2, or IGF2BP3 are found in Table 1. Scramble siRNA (siNC) for IGF2BP1, IGF2BP2, or IGF2BP3 was utilized as a negative control.

miR-26a-5p mimic (5′-UUCAAGUAAUCCAGGAUAGGCU-3′), miR-26a-5p inhibitor (5′-AGCCUAUCCUGGAUUACUUGAA-3′), and negative control (NC, 5′-CAGUACUUUUGUGUAGUACAA-3′) were synthesized by Genepharm Technologies (Shanghai, China). Transfections were performed utilizing Lipofectamine 2000 (Invitrogen) according to the manufacturer’s directions.

### Coculture assay

Coculture experiments were executed by seeding HNPC (5 × 10^4^ cells at the time of seeding) in a 12-well plate, and hucMSC cells (1 × 10^4^ cells) were grown on the Transwell (pore size, 0.4 µm; Merck, Millipore, Billerica, MA, USA). Cells were cocultured for 24 h and HNPC cells were then ready for further cytological experiments.

### Exosome experiments

Exosomes were purified by letting hucMSC cells reach 90% confluence, washing the cells with PBS, and incubating them with a newly prepared complete medium supplemented with exosome-free FBS for 48 h. The medium was then extracted and exosomes were collected by differential centrifugation. Apoptotic bodies and debris were depleted by centrifugation at 2,000 × *g* and 10,000 × *g* for 10 and 30 min, respectively. The exosomes were then harvested by centrifugation at 100,000 × *g* for 90 min. The exosome pellet was washed twice by resuspending in 20 mL PBS and ultracentrifugation at 100,000 × *g* for 90 min. The supernatant was filtered using a 0.22-mm filter (Steriflip, Millipore). Following centrifugation, exosomes were resuspended in PBS and subjected to ultracentrifugation as before. The supernatant was then removed, and exosomes were resuspended in 50 µL PBS.

Transmission electron microscopy (TEM) was executed by placing exosomes on a copper grid, excess exosomes wicked away, leaving a thin coat, and wetted with uranyl acetate (2%) in water. Grids were dried overnight, and the TEM was completed the following day. The size and concentration of exosome particles were determined with ZetaView Nanoparticle tracking system PMX 110 (Particle-Metrix, Meerbusch, Germany), and the data was processed using ZetaView 8.02.28.

Interactions between exosomes and HNPC were assayed as follows: the exosomes were stained using a PKH67 green fluorescent labeling kit (Sigma-Aldrich, St. Louis, MO, USA) according to the manufacturer’s protocol and cocultured with HNPC for 24 h. HNPC endocytosis of exosomes was assayed using a confocal microscope (Olympus FV1200, Japan).

### In vitro exosome transfer

To transfer exosomes, hucMSC were seeded at 500,000 cells per dish in exosome-free media and treated as indicated. Exosomes were harvested and reconstituted in 400 µL PBS. The HNPC were seeded into six-well plates and exposed to 100 µg/mL exosomes for 24 h.

### Cell viability assay

The HNPC viability was assessed using a Cell Counting Kit 8 (CCK8; Dojindo, Kumamoto, Japan). Briefly, HNPC after subsequent treatment were collected and pipetted into 96-well plates (100 µL; 1000 cells per well). To assess cell viability, 10 µL of CCK8 reagent was dispensed into every well followed by a 2-h incubation at 37 ℃. Absorption at 450 nm was measured via a microplate reader (BioTeck, San Jose, CA, USA).

### Cell death assay

Pyroptotic cell death was evaluated with active caspase-1 and propidium iodide (PI) staining. Briefly, the HNPC were seeded in six-well plates (5 × 10^5^ cells/well) and grown until reaching 50% confluence. Cells were then incubated with 660-YVAD-FMK (FLICA® 660 caspase-1 Assay Kit, ImmunoChemistry Technologie, Bloomington, MN, USA) per the protocol of the kit and stained with 10 μL PI (Thermo Fisher Scientific, Waltham, MA, USA) for 15 min followed by flow cytometry analysis (Beckman Coulter Cytoflex S, Beckman Coulter Life Science, Indianapolis, IN, USA).

### Measurement of lactate dehydrogenase

Following treatment, 5 × 10^5^ HNPC were seeded per well in a six-well plate and cultured for 24 h at 37 ℃. Cellular lactate dehydrogenase (LDH) activity was measured using the lactate dehydrogenase assay kit (A020-2-2; Nanjing Jiancheng Bioengineering Institute, Jiangsu, China) according to the kit manual.

### Enzyme-linked immunosorbent assay

The IL-1β and IL-18 abundance in HNPC supernatant was analyzed using commercial enzyme-linked immunosorbent assay (ELISA) kits (Human IL-1 beta Quantikine ELISA Kit, R&D Systems, Minneapolis, MN, USA, DLB50; IL-18 Human ELISA Kit, Thermo Fisher Scientific, KHC0181) according to the manufacturer’s directions, respectively. Optical density was evaluated employing a microplate reader at 450 nm.

### Luciferase reporter assay

METTL14 3′-UTR region carrying a putative miR-26a-5p binding site was inserted into the pGL3 vector. For METTL14 luciferase reporter assay, the HNPC were transfected with miR-26a-5p mimic and pGL3-METTL14-wild type (WT) or pGL3-METTL14-Mut plasmid (Mut) and pRL-TK vector (Promega, Madison, WI, USA) expressing the Renilla luciferase using Lipofectamine 2000. Otherwise, NLRP3 3′-UTR or 5′-UTR sequence was cloned into the pGl3 vector (Promega). HNPC transduced with METTL14 shRNA or overexpression vector were co-transfected with pGl3-NLRP3 3′-UTR or 5′-UTR luciferase reporter plasmid and pRL-TK vector (Promega) using Lipofectamine 2000 (Invitrogen). The relative luciferase activity was normalized to Renilla luciferase activity 48 h after transfection.

### m^6^A content analysis

Trizol reagent (Invitrogen) was used to extract the total RNA. Poly(A)^+^ RNA was purified using GenElute™ mRNA Miniprep Kit (Sigma, Louis, MO, USA). The m^6^A frequency in total RNA was assessed using the m6A RNA Methylation Assay Kit (Abcam, ab185912) per assay protocol. Briefly, 80 µL of binding solution and 200 ng of sample RNA were added into each designated well and then incubated at 37 ℃ for 90 min for RNA binding. Each well was washed thrice with wash buffer, added with 50 µL of the diluted capture antibody, and then incubated at room temperature for 60 min. Each well was subsequently incubated with detection antibody and enhancer solution at room temperature for 30 min. Finally, the wells were incubated with developer solution in the dark for 1–10 min at 25 ℃. The reaction was stopped with stop solution and determined using a microplate reader at 450 nm wavelength within 2–10 min.

### RNA immunoprecipitation assays

RNA immunoprecipitation (RIP) assays were executed using the Magna RIP RNA Binding Protein Immunoprecipitation kit (Millipore). Cells were prepared using RIP lysis buffer and the RNA–protein complexes were conjugated with anti-IGF2BP2 (Abcam, ab128175, 1:30) or anti-IgG antibody (Abcam, ab172730, 1:30) overnight at 4 ℃ and washed with RIP wash buffer for 10 min at 4 ℃ and then the RIP lysis buffer for 5 min at 4 ℃. The coprecipitated RNAs were purified using phenol/chloroform/isoamyl alcohol and subjected to qRT-PCR.

### mRNA stability measurement

Briefly, HNPC were treated with 0.2 mM actinomycin D for 30 min and harvested as 0 h conditions. The 0-, 4-, 8-, 12-, 16-, 20-, and 24-h samples were acquired, and RNA was extracted. cDNA synthesis by reverse transcriptase is primed by an oligo(dT) primer. mRNA levels were measured by qRT-PCR.

### RNA isolation and qRT-PCR

RNA from the HNP tissues and HNPC was obtained via TRIzol reagent (Invitrogen) extraction and RNeasy Mini Kit (Qiagen, Hilden, Germany) followed by reverse transcription using PrimeScript™ RT reagent kit with gDNA Eraser (Takara, Beijing, China). qRT-PCR was performed using SYBR® Premix Ex Taq™ GC (Takara). mRNA and miRNA expression was normalized to the internal control GAPDH or U6, respectively. qRT-PCR primers are found in Table 2.

**Table 2:** Primes sequences used in this study

Gene	Sequences (5′-3′)
METTL3-forward	CTCCACGCCAGATGCTCC
METTL3-reverse	GACAGTCCCTGCTACCTCCC
METTL14-forward	GGTTCTGGGGAGGGGTTG
METTL14-reverse	ATGAGGCAGTGTTCCTTTGTTC
WTAP-forward	GCACGCAGGGAAAACATCC
WTAP-reverse	ATAGTCCGACGCCATCAGG
NLRP3-forward	TTCGGAGATTGTGGTTGGG
NLRP3-reverse	TCAGGGAATGGCTGGTGC
IGF2BP1-forward	TCCCCGATGAGCAGATAGC
IGF2BP1-reverse	TTTGTGATGTTGCGGATGG
IGF2BP2-forward	TGGGAGGTGTTGGATGGAC
IGF2BP2-reverse	TCAAACTGATGCCCGCTTAG
IGF2BP3-forward	TCCCTACCCGCAGTTTGA
IGF2BP3-reverse	CGAGAAAGCTGCTTGATGTG
GAPDH-forward	AATCCCATCACCATCTTC
GAPDH-reverse	AGGCTGTTGTCATACTTC
miR-100-5p-forward	GCGAACCCGTAGATCCGAA
miR-100-5p-reverse	AGTGCAGGGTCCGAGGTATT
miR-21-5p-forward	GCGCGTAGCTTATCAGACTGA
miR-21-5p-reverse	AGTGCAGGGTCCGAGGTATT
miR-221-3p-forward	CGCGAGCTACATTGTCTGCTG
miR-221-3p-reverse	AGTGCAGGGTCCGAGGTATT
miR-26a-5p-forward	CGCGTTCAAGTAATCCAGGA
miR-26a-5p-reverse	AGTGCAGGGTCCGAGGTATT
miR-143-3p-forward	CGCGTGAGATGAAGCACTG
miR-143-3p-reverse	AGTGCAGGGTCCGAGGTATT
miR-27b-3p-forward	GCGCGTTCACAGTGGCTAAG
miR-27b-3p-reverse	AGTGCAGGGTCCGAGGTATT
U6-forward	CTCGCTTCGGCAGCACA
U6-reverse	AACGCTTCACGAATTTGCGT

### Western blot

Cell lysates were obtained using radioimmunoprecipitation assay lysis buffer (Beyotime, Shanghai, China) and measured via a bicinchoninic acid Protein Assay Kit (Beyotime). Approximately 20 µg of lysate was loaded per well on 10% sodium dodecyl sulfate–polyacrylamide gel electrophoresis gels followed by electronic transfer onto polyvinylidene fluoride membranes (Millipore), and 5% fat-free milk was used for blocking followed an overnight incubation at 4 ℃ with primary antibodies against METTL3 (ab195352; Abcam), METTL14 (ab220030; Abcam), WTAP (ab195380; Abcam), CD9 (ab223052; Abcam), CD63 (ab216130; Abcam), TSG101 (ab125011; Abcam), NLRP3 (ab263899; Abcam), caspase-1 (ab207802; Abcam), gasdermin-N domain (GSDMD-N; ab215203; Abcam), IGF2BP1 (ab82968; Abcam), IGF2BP2 (ab129071; Abcam), IGF2BP3 (ab177477; Abcam), and GAPDH (#5174, Cell Signaling Technology, Danvers, MA, USA). After antibody labeling, membranes were washed with Tris-Buffered Saline Tween-20 thrice times in total. Membranes were then hybridized with horseradish peroxidase-conjugated secondary antibodies (A0208, A0216; Beyotime). Protein was identified using an Enhanced Chemiluminescence Detection kit (Pierce Biotechnology, Rockford, IL, USA) and band illumination was quantified using Image-Pro Plus 6.0 software. GAPDH was used as a loading control.

### Statistical analysis

All statistical analyses were completed using GraphPad Prism 8.0.2 (GraphPad Software, San Diego, CA, USA). Data were presented as mean ± standard for three independent experiments. Two-sided Student’s *t-*test and analysis of variance were used to compare the variation among groups. *P* values < 0.05 were identified as significant.

## Results

### HucMSC promotes cell viability and inhibits expression of methyltransferases in HNPC

**Fig. 1:**
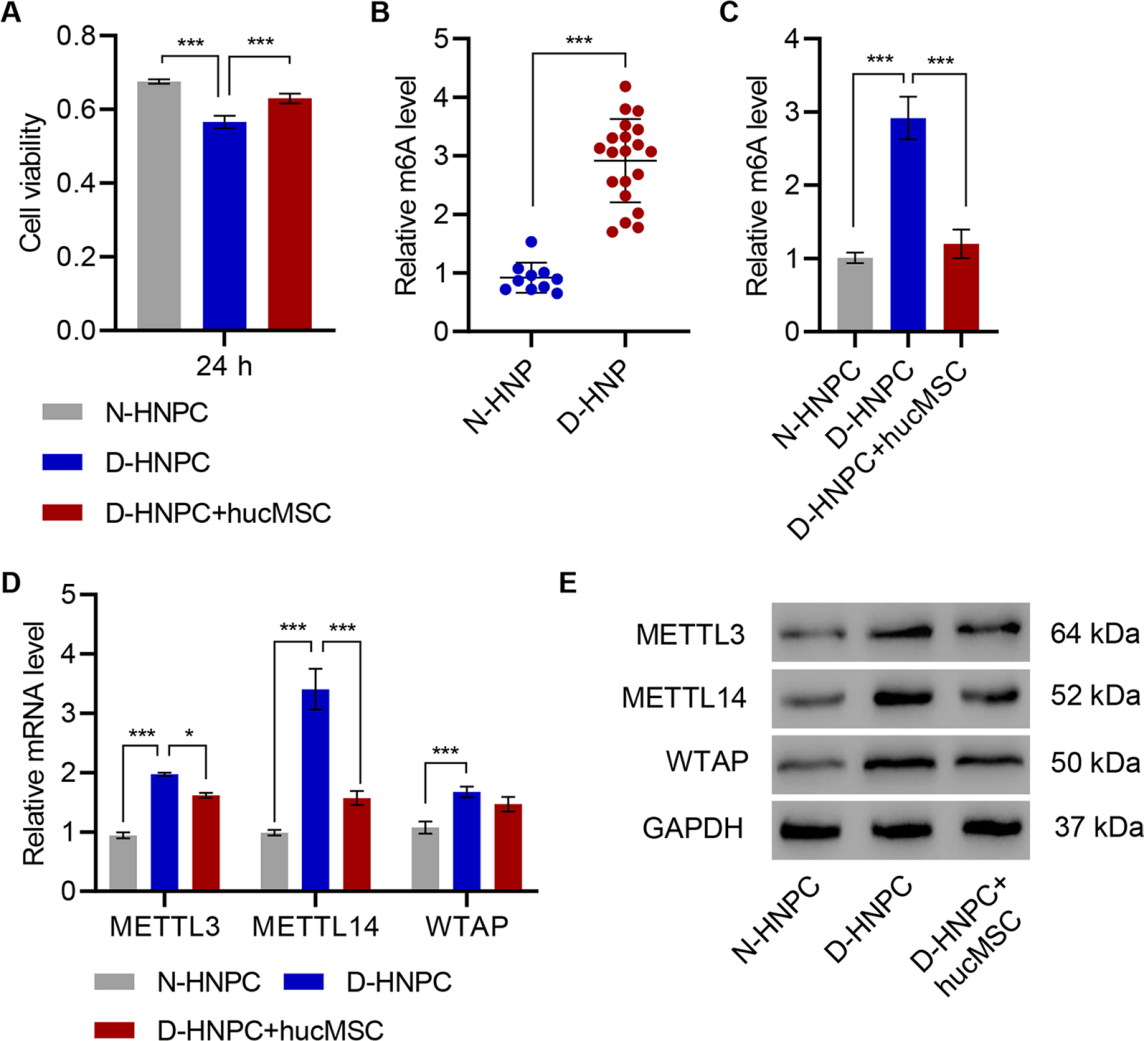
HucMSC promotes cell viability and inhibits the expression of methyltransferases in HNPC. **A** Cell viability and (**D**, **E**) expression of METTL3, METTL14, and WTAP in HNPC isolated from patients with intervertebral disc degeneration (D-HNPC) or normal controls (N-HNPC) with or without hucMSC coculture. **B**, **C** m^6^A levels in HNP tissues or HNPC isolated from patients with intervertebral disc degeneration (D-HNP; D-HNPC) or normal controls (N-HNP; D-HNPC). *P < 0.05, ***P < 0.001

Before addressing the function of MSCs, the hucMSC was first determined to continue to fulfill the minimal criteria for defining MSCs. First, the isolated hucMSC retained their morphology and capacity to adhere to plastic (Additional file 1: Figure S1A). Second, they maintained their surface markers, including the positive expressions of CD90, CD44, CD29, CD73, and CD105 and negative expressions of CD11b, CD14, CD34, CD45, and HLA-DR (Additional file 1: Figure S1B). Third, the hucMSC could readily be induced to differentiate into osteogenic and adipogenic lineages (Additional file 1: Figure S1C). To evaluate the potential beneficial effects of hucMSC on HNPC, a coculture system allowing the ready transfer of soluble factors from hucMSC to HNPC was employed. Compared to HNPC from normal donors (N-HNPC), the viability of HNPC donors from IVDD (D-HNPC) was dramatically reduced after 24 h in in vitro culture. However, their viability was partially restored when D-HNPC was cocultured with hucMSC (Fig. 1A). As the D-HNPC and hucMSC were not in physical contact, the hucMSC secretome was suggested to be responsible for the improvement in D-HNPC viability. Interestingly, m^6^A abundance was noticed to significantly increase in D-HNPC compared with N-HNPC as measured by methylation assay (Fig. 1B). Upon coculturing D-HNPC with hucMSC, m^6^A methylation was also suppressed to that of N-HNPC (Fig. 1C). Likewise, mRNA and protein of the m^6^A-mediating methyltransferases METTL3 and METTL14, as well as WTAP, were all enriched in D-HNPC compared with N-HNPC (Fig. 1D, E). The coculture of D-HNPC with hucMSC resulted in a significant decline in METTL14-relative mRNA and protein levels (Fig. 1D, E). Therefore, METTL14 was selected for further analysis to confirm its role in IVDD progression.

### HucMSC-derived exosomes promote cell viability and inhibit METTL14 expression in HNPC

**Fig. 2:**
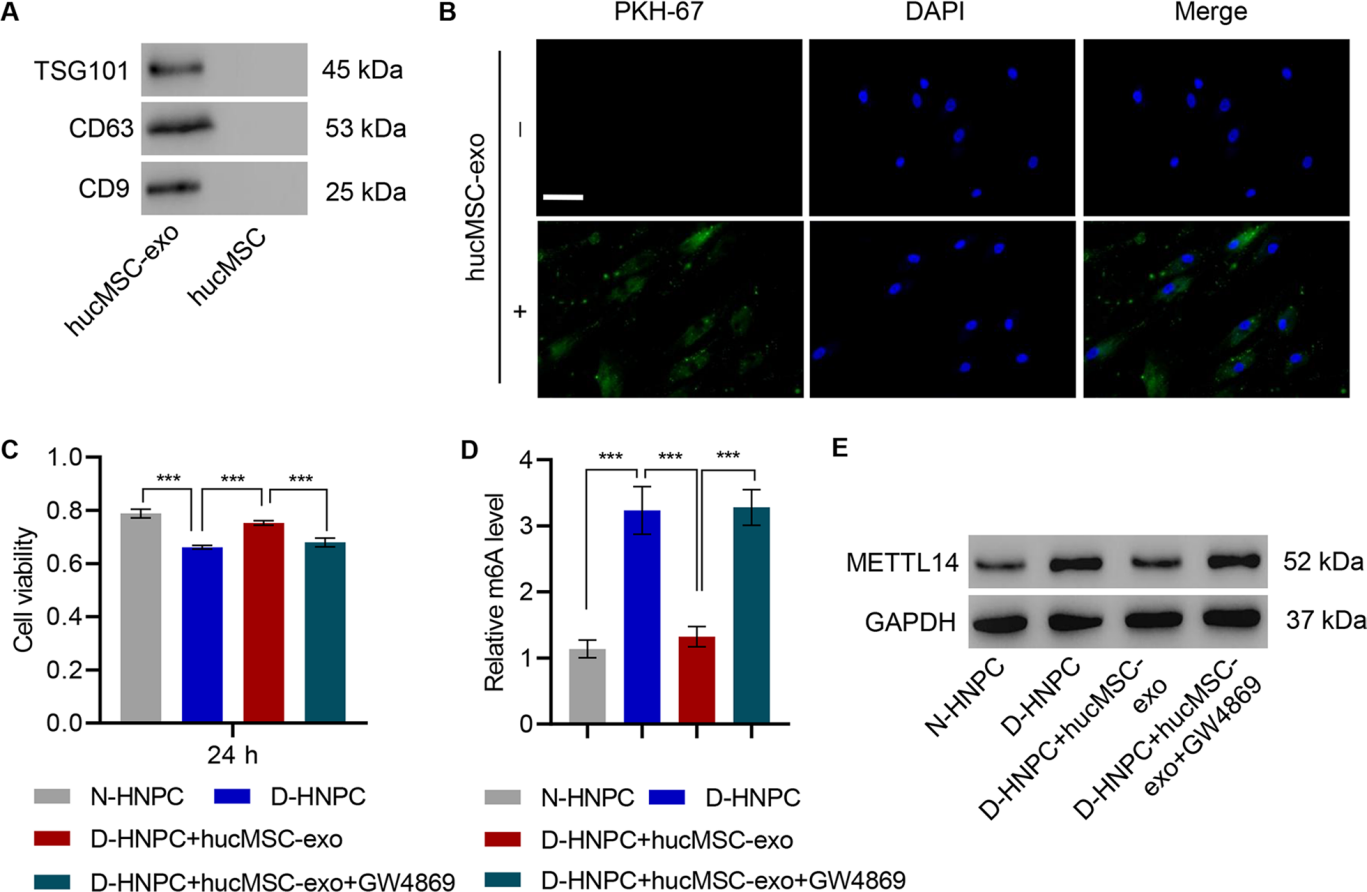
HucMSC-derived exosomes were successfully produced and endocytosed by HNPC. **A** Exosome markers CD63, CD9, and TSG101 proteins were detected by western blot in hucMSC-exo and hucMSC. **B** Internalization of exosomes by D-HNPC examined by laser scanning confocal microscope. *Scale bar*: 50 μm. Effect of hucMSC-exo depleted of exosomes on **C** cell viability, **D** m^6^A level, and **E** METTL14 expression in HNPC with or without 20 μM GW4869. ***P < 0.001

Since exosomes are an important bioactive component in the secretome of hucMSC, exosomes from a hucMSC-conditioned medium were purified to determine if exosomes mediated the hucMSC protective effects. Exosomes were successfully purified as evidenced by the expression of exosomal markers TSG101, CD63, and CD9 (Fig. 2A). The purified exosomes derived from hucMSC displayed as small round vesicles with an average diameter of either 65 ± 15 or 100 nm depending on the method utilized for particle size analysis (Additional file 1: Figure S2A–C). These results demonstrate that hucMSC can produce exosomes. Exosome endocytosis by D-HNPC was confirmed via PHK-67 staining and confocal microscopy (Fig. 2B). To validate that exosomes are responsible for the effects noted in the hucMSC coculture system, purified exosomes were administered to D-HNPC and viability assayed after 24-h incubation. Consistent with the previous observation, D-HNPC showed reduced viability compared with N-HNPC. However, D-HNPC treated with hucMSC-derived exosomes had significantly improved viability, paralleling the effects of coculture. The role of hucMSC exosomes was further confirmed by treating hucMSC with the exosome formation inhibitor GW4869 and administering the supernatant from these cells to D-HNPC. The supernatant provided no improvement in viability when compared to untreated D-HNPC (Fig. 2C).

Likewise, m^6^A abundance, which is enriched in D-HNPC, decreased to normal level when D-HNPC were treated with hucMSC exosomes. The supernatant from hucMSC treated with GW4869 does not effect on m^6^A abundance when compared with untreated D-HNPC (Fig. 2D). The same occurred for METTL14 expression, again confirming that exosomes are a primary active factor secreted from hucMSC (Fig. 2E).

### HucMSC-secreted exosomal miR-26a-5p targets METTL14 in HNPC

**Fig. 3:**
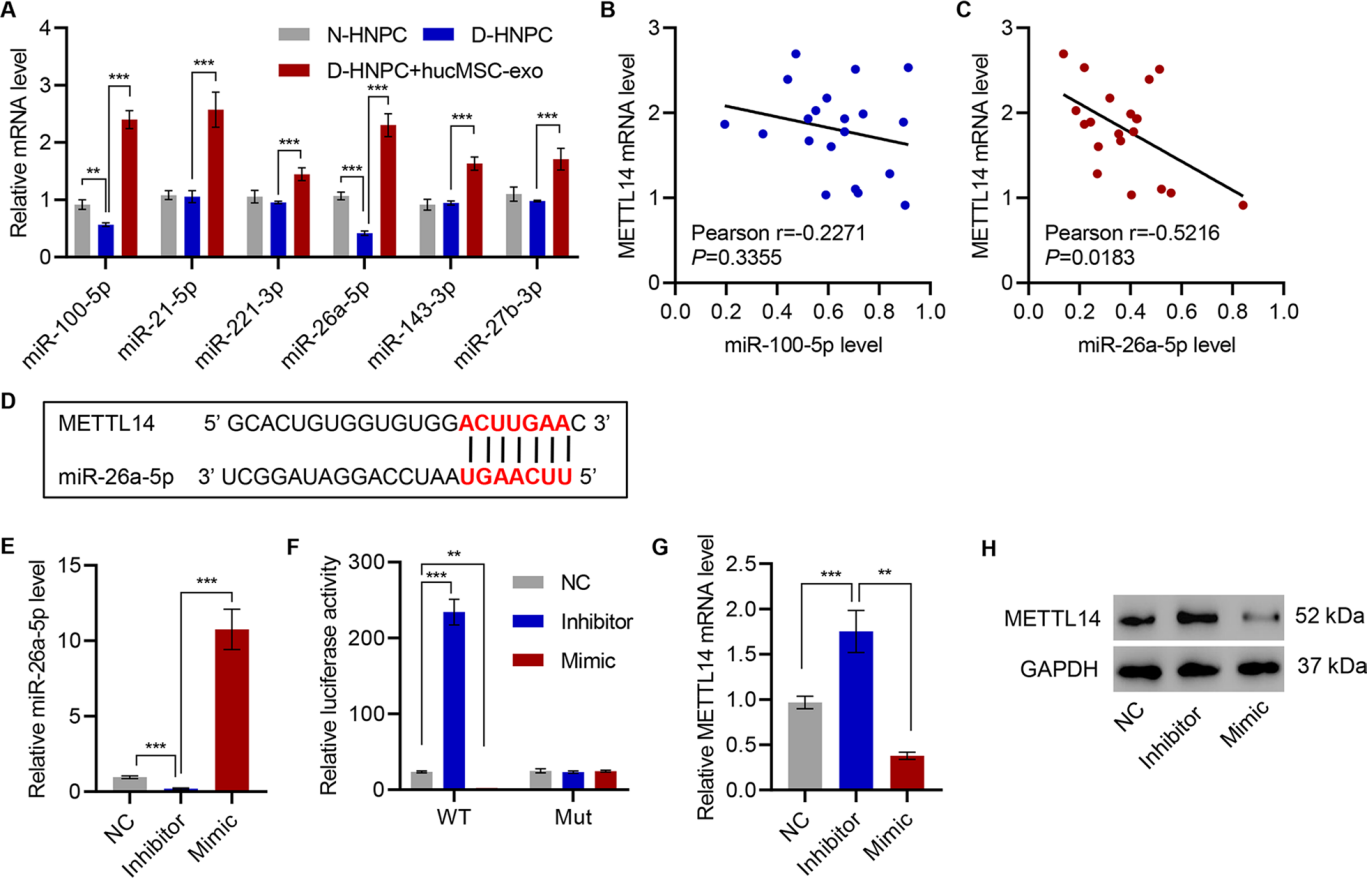
HucMSC-secreted exosomal miR-26a-5p targets METTL14 in HNPC. **A** Expression of miR-100-5p, miR-21-5p, miR-221-3p, miR-143-3p, and miR-27b-3p in HNPC with or without hucMSC coculture. **B**, **C** Pearson correlation scatter plots in D-HNP tissues (*n* = 20). **D** The predictive binding between miR-26a-5p and METTL14. **E**, **G**, **H** Relative expression of miR-26a-5p and METTL14 in D-HNPC transfected with miR-26a-5p mimic or its inhibitor. **F** Effect of miR-26a-5p mimic or its inhibitor on the luciferase activity of METTL14 3′-UTR wild type (WT) or mutant (Mut) in D-HNPC. **P < 0.01, ***P < 0.001

Since the hucMSC-derived exosomes simultaneously protected viability and downregulated METTL14 levels, exosomal microRNA may be responsible for such effects. Thus, a panel of microRNA that had previously been anti-apoptotic was examined (Huang et al. 2019; Su et al. 2019; Zhu et al. 2019; Wu et al. 2020). First, the abundance of miR-100-5p, miR-21-5p, miR-221-3p, miR-26a-5p, miR-143-3p, and miR-27b-3p were examined in N-HNPC, untreated D-HNPC, and D-HNPC treated with hucMSC exosomes. While all tested microRNA increased in D-HNPC following exosome treatment, only miR-100-5p and miR-26a-5p were reduced in D-HNPC when compared with N-HNPC (Fig. 3A). The miR-100-5p levels only showed weak correlation to METTL14 mRNA (*r* =  − 0.2271, *P* = 0.3355; Fig. 3B). However, miR-26a-5p presented with a moderate negative correlation (*r* =  − 0.5126) that reached statistical significance (*P* = 0.0183; Fig. 3C). Indeed, based on sequence complementariness, miR-26a-5p is predicted to bind to METTL14 mRNA (Fig. 3D).

miR-26a-5p was selected for further investigations because it decreased in D-HNPC, enriched with subsequent treatment by exosomes, and correlated to METTL14 mRNA level. A miR-26a-5p mimic and an antisense miR-26a-5p inhibitor were developed and verified that miR-26a-5p abundance was increased by D-HNPC transfection with the miR-26a-5p mimic and decreased by that of the inhibitor (Fig. 3E). D-HNPC were transfected with a METTL14 3′ UTR luciferase reporter or a mutant reporter in conjunction with either miR-26a-5p mimic or miR-26a-5p inhibitor. As predicted, the miR-26a-5p inhibitor induced an increase in luciferase activity, while the miR-26a-5p mimic decreased it. The mutant reporter was not affected by either treatment (Fig. 3F). Accordingly, both METTL14 mRNA and protein abundance were induced by the miR-26a-5p inhibitor and suppressed by the miR-26a-5p mimic (Fig. 3G, H).

### miR-26a-5p inhibitor diminished the pyroptosis inducing effects of METTL14 on HNPC

**Fig. 4:**
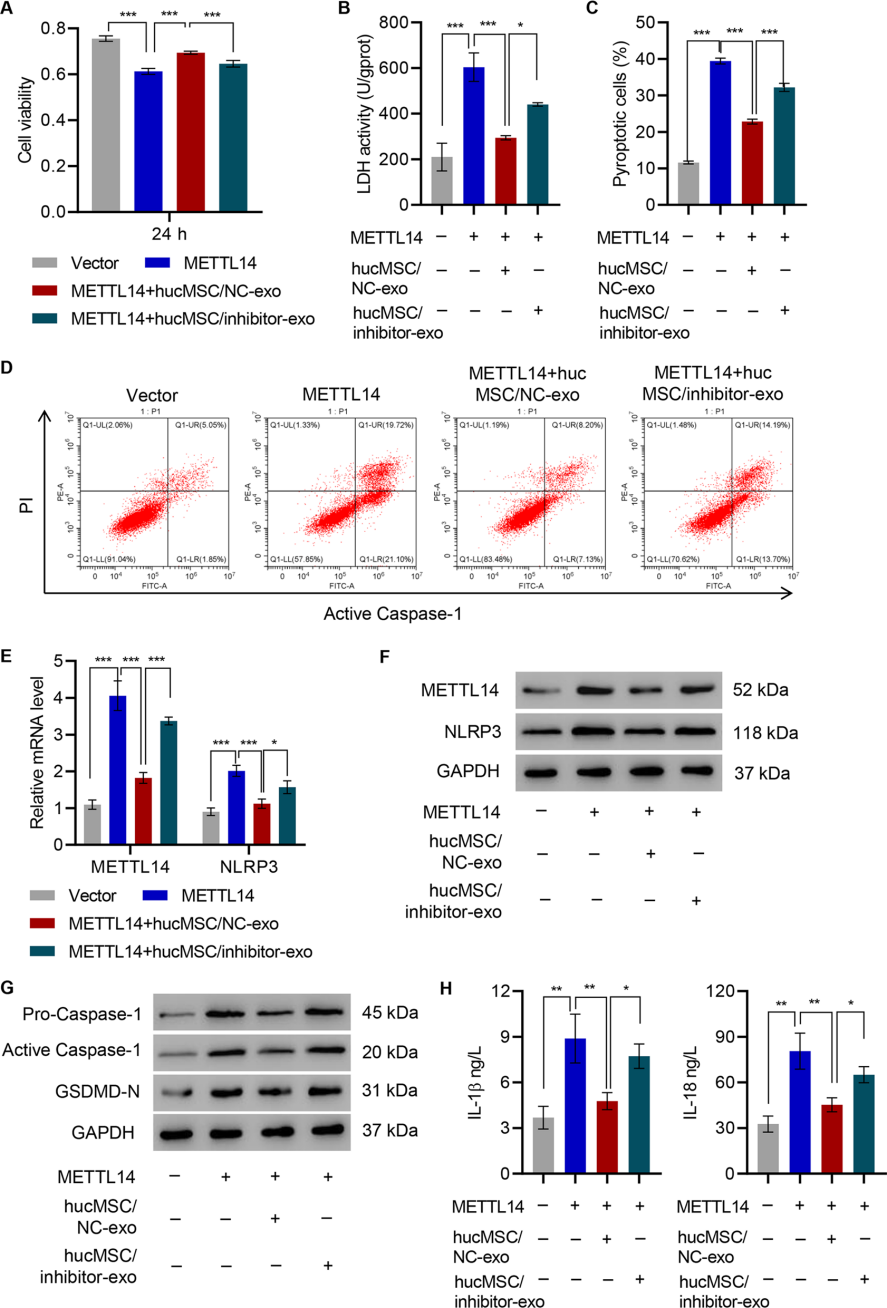
The effects of hucMSC on METTL14 overexpression on HNPC are inhibited by miR-26a-5p inhibitor. **A** Cell viability, **B** LDH activity, **C**, **D** pyroptosis, **E**–**G** expression of METTL14, NLRP3, caspase-1, and GSDMD-N, and **H** content of IL-1β and IL-18 were measured in D-HNPC transduced with the METTL14 expression vector and treated with hucMSC-exo from hucMSC transfected with miR-26a-5p inhibitor (hucMSC/inhibitor-exo) or NC (hucMSC/NC-exo). ***P < 0.001

D-HNPC with vectors overexpressing METTL14 was transfected to confirm the causal effects of METTL14 on HNPC cellular health (Additional file 1: Figure S3A). Indeed, HNPC transfected with METTL14 have reduced viability compared with those transfected with empty vectors (Fig. 4A). With the LDH-releasing assay, cell death induced by METTL14 reduced viability (Fig. 4B). To determine the types of cell death, flow cytometry analysis was conducted and it was found that more HNPC underwent pyroptosis upon METT14 transfection (Fig. 4C, D). As METTL14 is a direct target of miR-26a-5p, a miR-26a-5p inhibitor together with hucMSC exosomes was therefore supplied to evaluate its specific roles. Moreover, once miR-26a is significantly inhibited, it hampered the beneficial effects of hucMSC exosomes on HNPC, indicated by cell viability, LDH release, and the percentage of pyroptosis cells (Fig. 4A–D).

D-HNPC containing a METTL14 overexpression vector displayed an expected increase in METTL14 and NLPR3 expression. However, when METTL14-overexpressing D-HNPC were exposed to NC-transfected hucMSC exosomes, METTL14 and NLPR3 expressions were suppressed to nearly that of vector controls. Exosomes from hucMSC transfected with miR-26a-5p inhibitor significantly increased METTL14 and NLPR3 expressions (Fig. 4E, F). These data strongly suggest that the mechanism by which exosomes affect pyroptosis is dependent on METTL14 mRNA regulation.

Consistent with flow cytometry data, Western blot analysis of procaspase-1, activated caspase-1, and caspase-1 substrate GSDMD-N also confirmed that miR-26a could reduce METTL14-induced HNPC pyroptosis (Fig. 4G). Pyroptosis is induced by inflammatory cytokines (e.g., IL-1β and IL-18). The levels of these cytokines were next assayed by ELISA. The release of both cytokines was significantly increased following METTL14 transfection (Fig. 4H). The METTL14 impact on both pyroptotic cell death and the potential impact of compounding inflammation via cytokine release highlight this protein as a central player in IVDD pyroptosis. Importantly, miR-26a-5p, delivered by exosomes, demonstrates consistent suppression of not only pyroptosis but also the general inflammatory response underscores this as a potential mechanism to correct these aberrant pathways.

### NLRP3 as the downstream target of METTL14

**Fig. 5:**
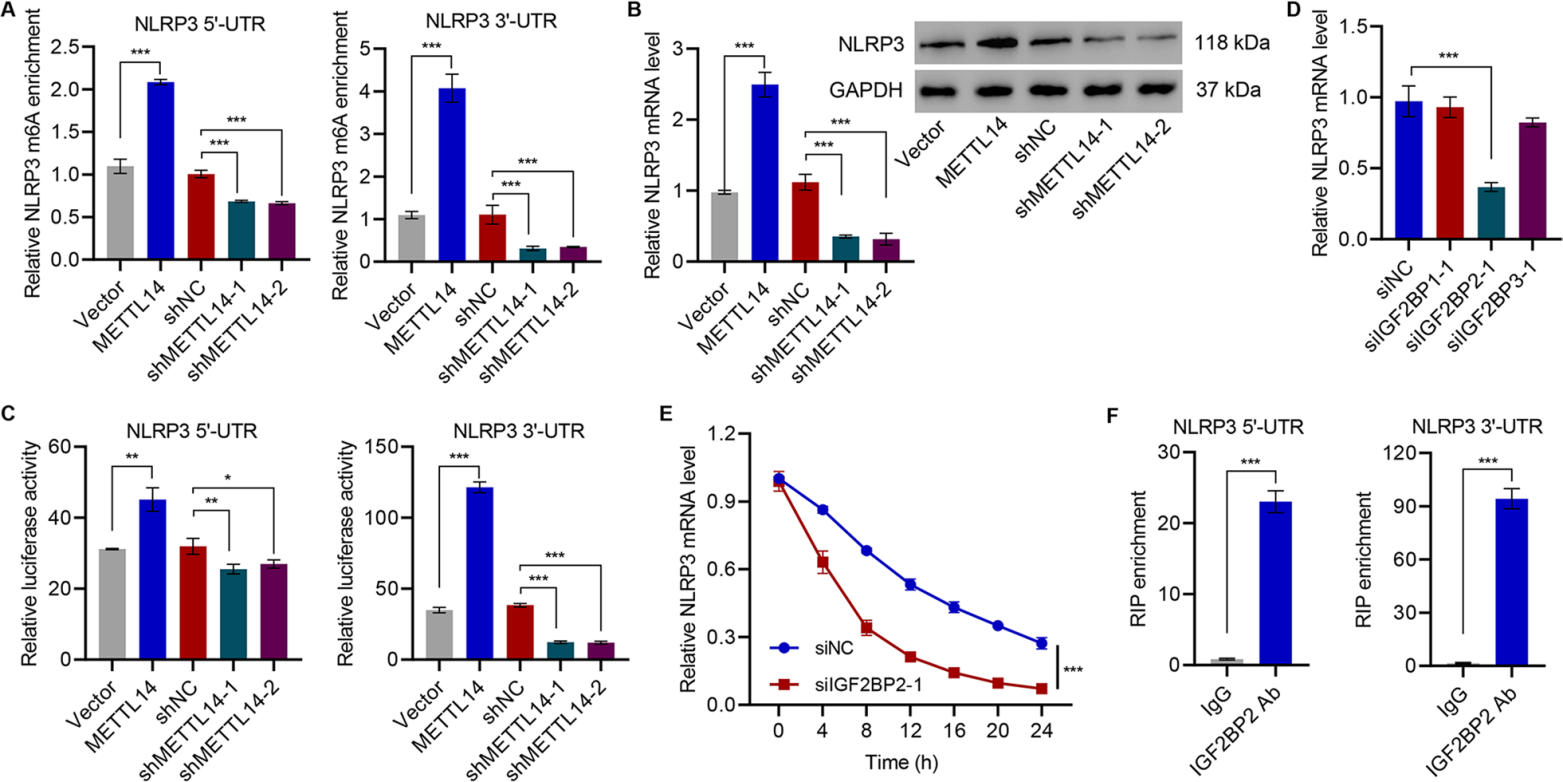
NLRP3 was identified as the target of METTL14. **A** MeRIP-qPCR analysis of NLRP3 5'-UTR and 3'-UTR m^6^A levels in D-HNPC transduced with METTL3 shRNA or expression vector. **B** Relative expression of NLRP3 in D-HNPC transduced with METTL14 shRNA or expression vector was detected by qRT-PCR or western blot. **C** Luciferase activity was measured in D-HNPC transduced with METTL14 shRNA or expression vector. **D** The NLRP3 mRNA level was quantified by qRT-PCR in D-HNPC transfected with IGF2BP1, IGF2BP2, or IGF2BP3 siRNA. **E** The NLRP3 transcript was measured by qRT-PCR in D-HNPC transfected with IGF2BP2 siRNA. **F** The binding of IGF2BP2 to NLRP3 mRNA was measured by RIP and qRT-PCR

As the NLRP3 inflammasome plays a central role in IVDD in addition to its correlation to METTL14 and response to exosome treatment, the current study sought to determine its relationship to METTL14, which has been shown to increase m^6^A abundance. Thus, the current study aimed to clarify whether METTL14 affects NLRP3 via m^6^A methylation. Upon METTL14 transfection, the m6A levels in both 3′ and 5′ UCR were dramatically increased (Fig. 5A). While METTL14 shRNA reduced the m6A levels of NLRP3 (Figure S3B, 5A), METTL14 mediated the m^6^A methylation of NLRP3 mRNA. Such modification increased the cellular levels of NLRP3 in HNPCs and resulted in abundant NLRP3 protein translation (Fig. 5B).

Moreover, cells expressing either a 3′ UTR NLRP3 or a 5′ UTR NLRP3 luciferase reporter showed increased luciferase activity in METTL14-overexpressing cells. Both reporters demonstrated a decrease in activity following METTL14 knockdown confirming a role for METTL14 in regulating NLRP3 protein presence via mRNA (Fig. 5C). As METTL14 expression increased NLRP3 protein through an increase in mRNA levels and specifically induced an increase in NLRP3 mRNA m^6^A, this study sought to determine if the mechanism was through mRNA stabilization via the known m^6^A chaperones including IGF2BP1, IGF2BP2, and IGF2BP3. Indeed, siRNA directed at the m^6^A-targeting IGF2BP2 produced a rapid decrease in NLRP3 mRNA levels over a 6-h time course while siRNA directed at IGF2BP1 or IGF2BP3 did not significantly alter NLRP3 mRNA levels (Figs. 5D, E and S4A–C). On performing RIP using an antibody targeting IGF2BP2, the presence of both 3′ UTR and 5′ UTR of NLRP3 mRNA as measured by qRT-PCR was found, demonstrating the direct binding of IGF2BP2 to NLRP3 mRNA. This strongly suggests that METTL14-induced m^6^A methylation of NLRP3 mRNA recruits IGF2BP2 to increase mRNA stability (Fig. 5F).

### The effects of METTL14 silencing on HNPC inhibited by NLRP3 overexpression

**Fig. 6:**
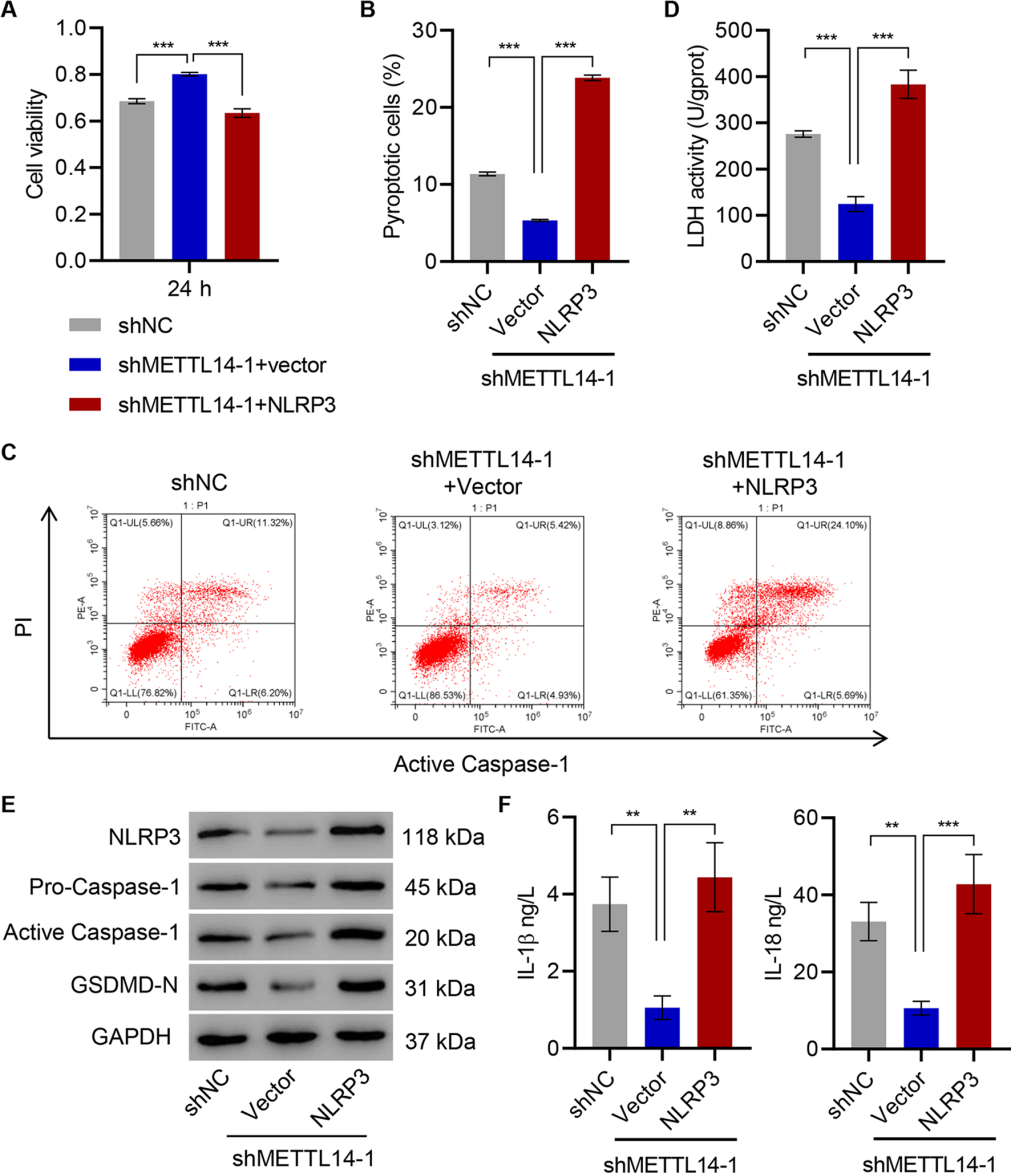
The effects of METTL14 silencing on HNPC are inhibited by NLRP3 overexpression. **A** Cell viability, **B**, **C** pyroptosis, **D** LDH activity, **E** expression of NLRP3, caspase-1, and GSDMD-N, and **F** content of IL-1β and IL-18 were measured in D-HNPC transduced with METTL14 shRNA vector and NLRP3 expression vector. **P < 0.01, ***P < 0.001

The critical roles of METTL14 in regulating NLRP3 and pyroptosis in D-HNPC were identified, and elucidating the role of the relationship between METTL14 and NLRP3 in pyroptosis was next sought. The impact of METTL14 silencing on D-HNPC was measured with and without NLRP3 overexpression (Figure S3C). While the METTL14 knockdown increased cell viability, NLRP3 overexpression reduced it to that of cells treated with control shRNA (Fig. 6A). Likewise, the proportion of pyroptotic cells decreased from a baseline of approximately 10% in control shRNA-transfected cells to approximately 5% following METTL14 knockdown (Fig. 6B, C). NLRP3 overexpression superimposed on METTL14 knockdown increased in pyroptotic cells as measured by active caspase-1 and PI staining double-positive to approximately 25% (Fig. 6B, C). A similar pattern was noted with LDH release wherein METTL14 suppression decreased LDH activity and METTL14 shRNA plus NLRP3 overexpression resulted in a net increase in LDH activity over shRNA alone or NC (Fig. 6D). Procaspase-1, active caspase-1, and GSDMD-N followed a similar pattern of decrease with METTL14 knockdown and complete restoration when NLRP3 was overexpressed (Fig. 6E). Importantly, both IL-1β and IL-18 secretion was decreased from METTL14 shRNA-expressing cells and were both fully rescued with forced expression of NLRP3 (Fig. 6F). The pyroptotic effects of METTL14 induced in D-HNPC are mediated through NLRP3.

### The expression of NLRP3 correlates positively with METTL14 and IGF2BP2 and negatively with miR-26a-5p

**Fig. 7:**
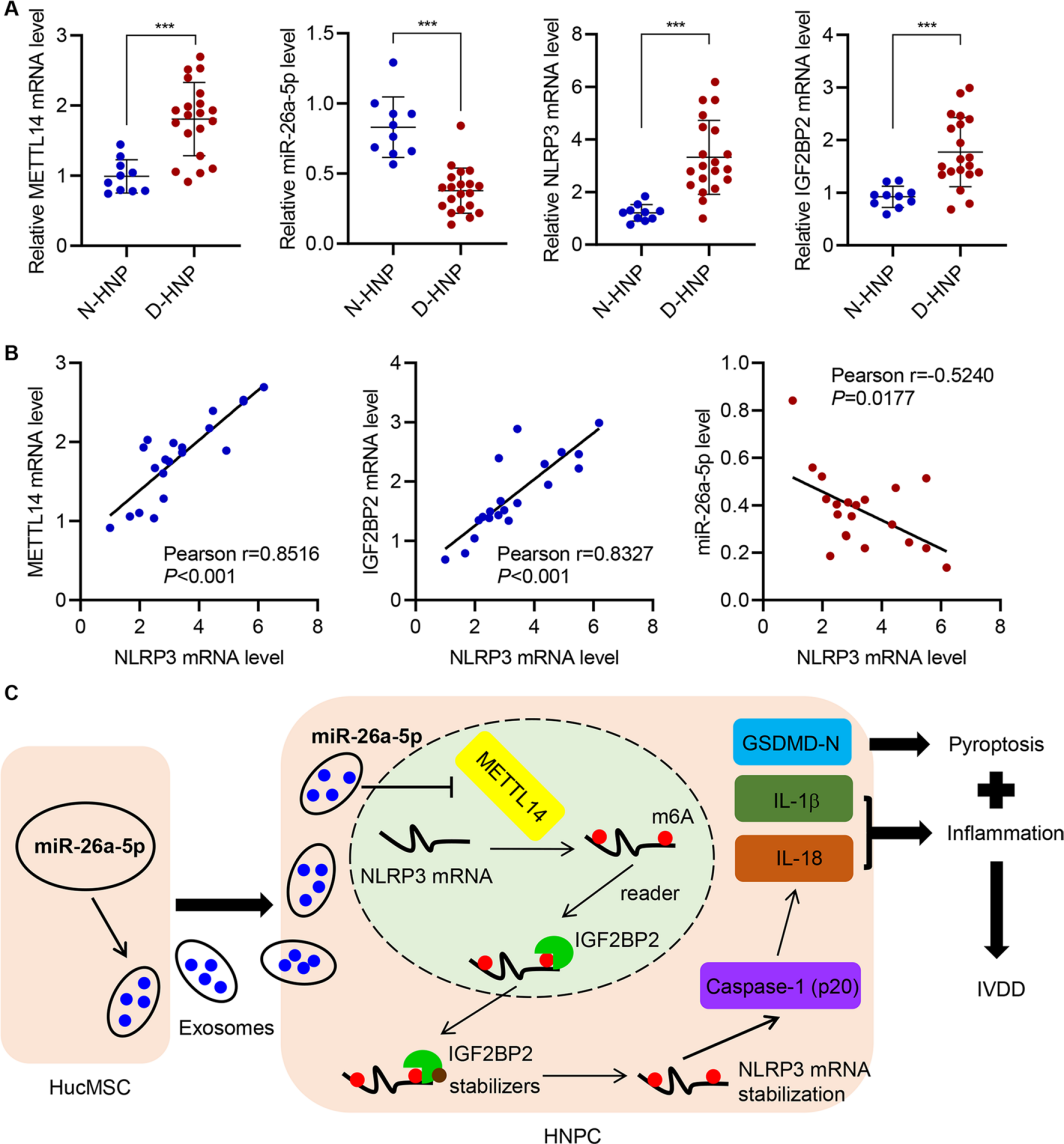
The expression of NLRP3 correlates positively with METTL14 and IGF2BP2 and negatively with miR-26a-5p. **A** qRT-PCR analysis of METTL14, miR-26a-5p, NLRP3, and IGF2BP2 in N-HNP tissues (*n* = 10) and D-HNP tissues (*n* = 20). **B** Pearson correlation analysis among METTL14, miR-26a-5p, NLRP3, and IGF2BP2 level in 20 cases of D-HNP tissues. **C** Schematic representation of the regulation of HNPC inflammation and pyroptosis by hucMSC-secreted exosomal miR-26a-5p/METTL14/NLRP3/caspase-1. ***P < 0.001

With the detailed molecular pathway of METTL14/NLRP3-mediated pyroptosis, the broader clinical pertinence of these was examined by comparing mRNA from N-HNP and D-HNP tissues. For this, 10 N-HNP and 20 D-HNP samples were assayed using qRT-PCR and/or Western blot for expression of METTL14, miR-26a-5p, NLRP3, and IGF2BP2. Each of which was significantly different between the two tissues with miR-26a-5p reduced in the D-HNP samples and METTL14, NLRP3, and IGF2BP2 all increased, showing that the relationships between these are applicable in a broader clinical setting (Figs. 7A and Additional file 1: Figure S5). To determine potential causal relationships, Pearson *R*^2^ correlation values for each component against NLRP3 expression. METTL14 and IGF2BP2 mRNA were strongly positively correlated to NLRP3 mRNA, supporting a closely linked relationship (such as m^6^A methylation) among these groups, while miR-26a-5p was weakly negatively correlated to NLRP3 expression (Fig. 7B). Based on the available data thus far, a model in which miR-26a-5p can moderate the IVDD effects via a METTL14/NLRP3 inflammatory pathway was proposed (Fig. 7C).

## Discussion

This study demonstrated the applicability of using human MSCs to supply miR-26a-5p via exosomes to inhibit the METTL14/NLRP3 pathway to prevent pyroptosis in NP cells. Mechanistically, the current study found that miR-26a-5p, contained in these exosomes, blunts the expression of the mRNA methyltransferase METTL14. The loss of METTL14, in turn, reduces m^6^A methylation of NLRP3 mRNA. m^6^A methylation of NLRP3 allows IGF2BP2 to bind to the mRNA, stabilize it, and thus increase the level of NLRP3 protein. As NLRP3 is a well-known inflammasome component, it is not surprising that the loss of NLRP3, either through direct targeting or through upstream alteration, results in a decrease in both pyroptosis and a reduction in proinflammatory cytokines. Indeed, a reduced NLRP3 presence as a downstream effect of miR-26a-5p targeting METTL14 lessens inflammatory cell death (pyroptosis). NLRP3 is a critical component of an inflammasome responsible for pyroptosis and its expression is directly correlated to a host of inflammatory cell death responses (e.g., IL-1β and IL-18 secretion and caspase-1 expression/activation). As inflammation is well documented in IVDD (Johnson et al. 2015; Zhang et al. 2017; Cunha et al. 2018; Li et al. 2018), a central function for NLRP3 is expected. In another study, upregulation of NLRP3 in an animal IVDD model with accompanying increases on inflammatory IL-β release (Chen et al. 2020).

The role of m^6^A modification is particularly noteworthy as it involves the *writer* complex consisting of active METTL3, supportive METTL14, and regulatory WTAP (Bokar et al. 1997; Ping et al. 2014; Wang et al. 2016) and has been associated with inflammation in other conditions including ischemia/reperfusion injury (Diao et al. 2020) and T regulatory cell function (Tong et al. 2018; Wang et al. 2020a). While little research has previously been completed on the impact of m^6^A on IVDD, strong research into modulating m^6^A modification via small molecules has increased (Gu et al. 2020). Overall, this presents another opportunity to develop an IVDD treatment as this is a cornerstone of the pathway modulated by exosome treatment. Further studies are needed to confirm the appropriateness of small molecular modulators of m^6^A frequency as IVDD therapeutics and the capability of m^6^A readers beyond IGF2BP2 in this context.

Possibly the most important finding of the current study is the efficacy of exosome-administered miR-26a-5p. Reduced in D-HNPC, this microRNA can be effectively administered using exosomes produced by hucMSC not only producing a molecular effect by reducing both METTL14 and NLRP3 presence but reducing inflammatory conditions via suppressing cytokine release and finally lowering proptosis frequency itself. A previous study found that miR-26a-5p is upregulated in the mice serum with IVDD and postulated that this increase exacerbated the disease (Fan et al. 2019), which is not in agreement with the current finding. The current paper proposed that this may be due to the limitations of a murine model previously used compared with the human tissue used here or the possibility that the increase in serum, rather than tissue, microRNA concentration may be an adaptive attempt by the body to promote tissue repair at the disk site. Therefore, the potential role of miR-26a-5p in IVDD progression should be further discussed. The potential role of exosomes in IVDD treatment is of particular importance. Although the models and depth of molecular examination vary by study, robust research highlighting the capability of exosome treatment has been completed (Liao et al. 2019; Xia et al. 2019). Indeed, clinical therapy can be developed with a thorough understanding of both the effectiveness and the mechanism of the action of exosomes.

As pyroptosis is a central IVDD feature, and these developments may be incorporated as a direct treatment option using exosomes directly to reduce inflammatory cell death. Additionally, several important participants involved in pyroptosis (NLRP3, METTL14, and IGF2BP2) have been positively identified, these may be targeted independently using small molecules or gene therapy for IVDD treatment.

Additional potential areas of study, outside of this current work, include an examination of other components of exosomes produced by hucMSC, as well as direct administration of exosomes, small molecules, or other therapeutics to target the pathways discussed here. IVDD is a complicated disease with multiple overlaying factors contributing to tissue damage. It remains unclear if the pathway described here interacts with additional factors or how many processes operate in parallel to induce pyroptosis. Accordingly, whether other inflammasomes such as NLPR1, NLR family CARD domain containing 4 (NLRC4), or absent in melanoma 2 (AIM2) participate in IVDD pyroptosis remains to be determined. Another limitation of this study was the lack of animal models, which would highlight the therapeutic effects of hucMSC-exo on IVDD. Therefore, the animal model experiment would be further performed.

## Conclusions

The results of this study highlight the capability of exosomes in the treatment of patients with IVDD. The current study identified the active component of exosomes (miR-26a-5p) and the downstream targets of this microRNA, thus leading to the development of numerous therapeutic approaches based on this research. Further studies are needed to ascertain if direct treatment with exosomes or a directed approach targeting downstream proteins is more efficacious.

## Supplementary Information

**Additional file 1: Table S1.** Human IVDD specimen information. Figure S1. Characterization and differentiation assay of hucMSC. (A) HucMSC cultured in normal cell culture medium (Passage 3). (B) HucMSC were stained positive with CD29, CD90, CD44, CD73, and CD105 antibodies, negative with CD11b, CD14, CD34, CD45, and HLA-DR antibody, which was identified on the using flow cytometry. (C) Adipogenic (left) and osteogenic (right) differentiation of hucMSC assessed by Oil red O staining and Alizarin Red staining, respectively. Scale bar = 100 μm. **Figure S2**. Characterization of exosomes from HucMSC. (A) Transmission electron micrograph of hucMSC-derived exosomes (hucMSC-exo). Scale bar: 200 nm. (B) Nanoanalyzer analysis of particle size distribution of exosomes, and the average diameter of exosomes was 65 ± 15 nm. (C) Particle size distribution in purified pellets consistent with size range of exosomes (average size 100 nm), measured by ZetaView® Particle Tracking Analyzer. **Figure S3. **Expression of METTL14 and NLRP3 in HNPC. (A-C) Relative expression of METTL14 and NLRP3 in HNPC transduced with METTL14 shRNA vector, METTL14 overexpression vector, or NLRP3 overexpression vector. ***P<0.001. **Figure S4.** Expression of IGF2BP1, IGF2BP2 and IGF2BP3 in HNPC. (A-C) Relative expression of IGF2BP1, IGF2BP2, and IGF2BP3 in HNPC transfected with IGF2BP1, IGF2BP2, or IGF2BP3 siRNA. ***P<0.001.**Figure S5. **Expression of METTL14, NLRP3, and IGF2BP2 in HNPC. Western blot analysis of METTL14, NLRP3, and IGF2BP2 in N-HNP tissues (n = 10) and D-HNP tissues (n = 20).

## Data Availability

The authors confirm the availability of all data generated or analyzed in this manuscript.
