# Supplementary material for: Human umbilical cord mesenchymal stem cells deliver exogenous miR-26a-5p via exosomes to inhibit nucleus pulposus cell pyroptosis through METTL14/NLRP3
Source: Mol Med. 2021 Aug 19;27:91. doi: 10.1186/s10020-021-00355-7 (PMC8375162; doi:10.1186/s10020-021-00355-7)
Supplement: Supplementary file 1 — Additional file 1: Table S1. Human IVDD specimen information. Figure S1. Characterization and differentiation assay of hucMSC. (A) HucMSC cultured in normal cell culture medium (Passage 3). (B) HucMSC were stained positive with CD29, CD90, CD44, CD73, and CD105 antibodies, negative with CD11b, CD14, CD34, CD45, and HLA-DR antibody, which was identified on the using flow cytometry. (C) Adipogenic (left) and osteogenic (right) differentiation of hucMSC assessed by Oil red O staining and Alizarin Red staining, respectively. Scale bar = 100 μm. Figure S2. Characterization of exosomes from HucMSC. (A) Transmission electron micrograph of hucMSC-derived exosomes (hucMSC-exo). Scale bar: 200 nm. (B) Nanoanalyzer analysis of particle size distribution of exosomes, and the average diameter of exosomes was 65 ± 15 nm. (C) Particle size distribution in purified pellets consistent with size range of exosomes (average size 100 nm), measured by ZetaView® Particle Tracking Analyzer. Figure S3. Expression of METTL14 and NLRP3 in HNPC. (A-C) Relative expression of METTL14 and NLRP3 in HNPC transduced with METTL14 shRNA vector, METTL14 overexpression vector, or NLRP3 overexpression vector. ***P<0.001. Figure S4. Expression of IGF2BP1, IGF2BP2 and IGF2BP3 in HNPC. (A-C) Relative expression of IGF2BP1, IGF2BP2, and IGF2BP3 in HNPC transfected with IGF2BP1, IGF2BP2, or IGF2BP3 siRNA. ***P<0.001.Figure S5. Expression of METTL14, NLRP3, and IGF2BP2 in HNPC. Western blot analysis of METTL14, NLRP3, and IGF2BP2 in N-HNP tissues (n = 10) and D-HNP tissues (n = 20). [file 10020_2021_355_MOESM1_ESM.docx]

**Human** **umbilical cord mesenchymal stem cells deliver exogenous miR-26a-5p via exosomes to inhibit nucleus pulposus cell pyroptosis through METTL14/NLRP3**

Xiaoqiu Yuan*, Tiefeng Li*, Lei Shi, Jinhao Miao, Yongfei Guo, Yu Chen

Spine Center, Department of Orthopaedics, Changzheng Hospital, Naval Medical University, Shanghai 200003, China

* Contributed equally

Corresponding Author’s information: Yu Chen, Spine Center, Department of Orthopaedics, Changzheng Hospital, Naval Medical University, No 415 Fengyang Road, Shanghai 200003, China. Tel: +86-02181885792, E-mail: [cyspine@smmu.edu.cn](mailto:cyspine@smmu.edu.cn)

**Running title:** miR-26a-5p/METTL14/NLRP3 axis in intervertebral disc degeneration

**Table S1. Human IVDD specimen information**

| **Donor** | **IVD level** | **Age** | **Sex** | **Disease** | **Symptoms** | **Duration of symptoms prior to surgery** | **Pfirrmann Grade** |
| --- | --- | --- | --- | --- | --- | --- | --- |
| 1 | L5-L6 | 53 | M | IVDD | Radiculopathy: LBP, LP and palsy | 6 months | III |
| 2 | L5-L6 | 46 | F | IVDD | Radiculopathy: LBP, LP and palsy | 10 months | III |
| 3 | L5-L6 | 58 | F | IVDD | Radiculopathy: LBP, LP and palsy | 8 months | III |
| 4 | L5-L6 | 49 | M | IVDD | Radiculopathy: LBP, LP and palsy | 12 months | III |
| 5 | L5-L6 | 55 | F | IVDD | Radiculopathy: LBP, LP and palsy | 9 months | IV |
| 6 | L5-L6 | 62 | F | IVDD | Radiculopathy: LBP, LP and palsy | 14 months | IV |
| 7 | L5-L6 | 52 | F | IVDD | Radiculopathy: LBP, LP and palsy | 24 months | IV |
| 8 | L4-L5 | 60 | M | IVDD | Radiculopathy: LBP, LP and palsy | 10 months | IV |
| 9 | L5-L6 | 58 | M | IVDD | Radiculopathy: LBP, LP and palsy | 14 months | IV |
| 10 | L4-L5 | 54 | F | IVDD | Radiculopathy: LBP, LP and palsy | 40 months | V |
| 11 | L4-L5 | 45 | M | IVDD | Radiculopathy: LBP, LP and palsy | 18 months | V |
| 12 | L4-L5 | 52 | M | IVDD | Radiculopathy: LBP, LP and palsy | 21 months | V |
| 13 | L5-L6 | 49 | F | IVDD | Radiculopathy: LBP, LP and palsy | 18 months | V |
| 14 | L5-S1 | 43 | M | IVDD | Radiculopathy: LBP, LP | 12 months | III |
| 15 | L5-S1 | 48 | M | IVDD | Radiculopathy: LBP, LP | 8 months | III |
| 16 | L5-S1 | 59 | M | IVDD | Radiculopathy: LBP, LP | 9 months | III |
| 17 | L5-S1 | 66 | F | IVDD | Radiculopathy: LBP, LP | 10 months | IV |
| 18 | L5-S1 | 61 | F | IVDD | Radiculopathy: LBP, LP | 14 months | IV |
| 19 | L5-S1 | 53 | F | IVDD | Radiculopathy: LBP, LP | 24 months | IV |
| 20 | L4-L5 | 68 | M | IVDD | Radiculopathy: LBP, LP | 21 months | V |
| 21 | T12-L1 | 16 | F | IS | Dyskinesia | 16 months | I |
| 22 | T12-L1 | 20 | F | IS | Dyskinesia | 16 months | I |
| 23 | T12-L1 | 18 | M | IS | Dyskinesia | 16 months | I |
| 24 | L1-L2 | 22 | F | IS | Paresthesia | 10 months | I |
| 15 | L1-L2 | 18 | M | IS | Paresthesia | 10 months | I |
| 26 | L1-L2 | 18 | M | FTLF | Myelopathy: pain and palsy | 1 day | I |
| 27 | L1-L2 | 20 | M | FTLF | Myelopathy: pain and palsy | 1 day | I |
| 28 | L1-L2 | 22 | F | FTLF | Myelopathy: pain and palsy | 1 day | I |
| 29 | L2-L3 | 20 | F | FTLF | Paresthesia, dyskinesia | 1 day | I |
| 30 | L2-L3 | 22 | F | FTLF | Paresthesia, dyskinesia | 1 day | I |

F, female; FTLF, fresh traumatic lumbar fractures; IS, Idiopathic scoliosis; IVD, intervertebral disc; IVDD, intervertebral disc degeneration; M, male; L, lumbar; LBP, low back pain; LP, leg pain.


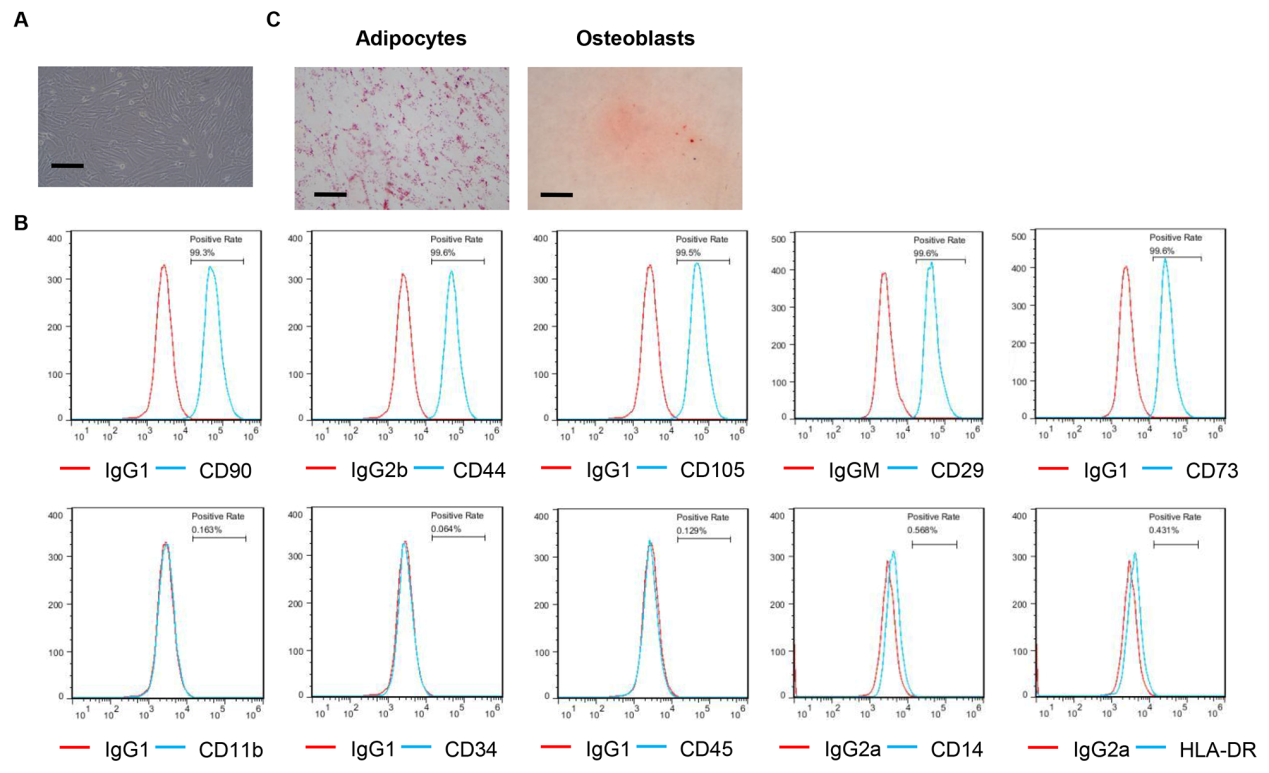


**Figure S1. Characterization and differentiation assay of hucMSC.** (A) HucMSC cultured in normal cell culture medium (Passage 3). (B) HucMSC were stained positive with CD29, CD90, CD44, CD73, and CD105 antibodies, negative with CD11b, CD14, CD34, CD45, and HLA-DR antibody, which was identified on the using flow cytometry. (C) Adipogenic (left) and osteogenic (right) differentiation of hucMSC assessed by Oil red O staining and Alizarin Red staining, respectively. Scale bar = 100 μm.


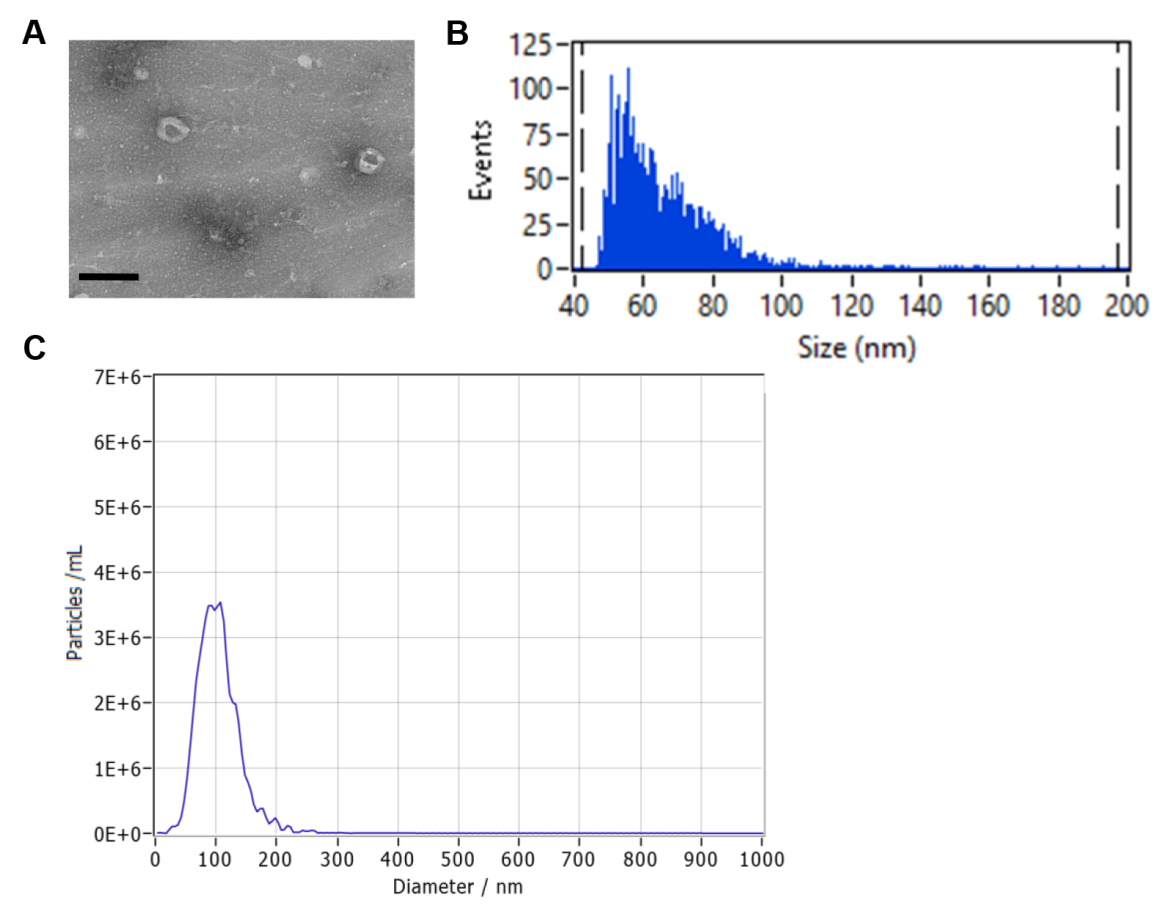


**Figure S2.** **Characterization of exosomes from HucMSC**. (A) Transmission electron micrograph of hucMSC-derived exosomes (hucMSC-exo). Scale bar: 200 nm. (B) Nanoanalyzer analysis of particle size distribution of exosomes, and the average diameter of exosomes was 65 ± 15 nm. (C) Particle size distribution in purified pellets consistent with size range of exosomes (average size 100 nm), measured by ZetaView® Particle Tracking Analyzer.


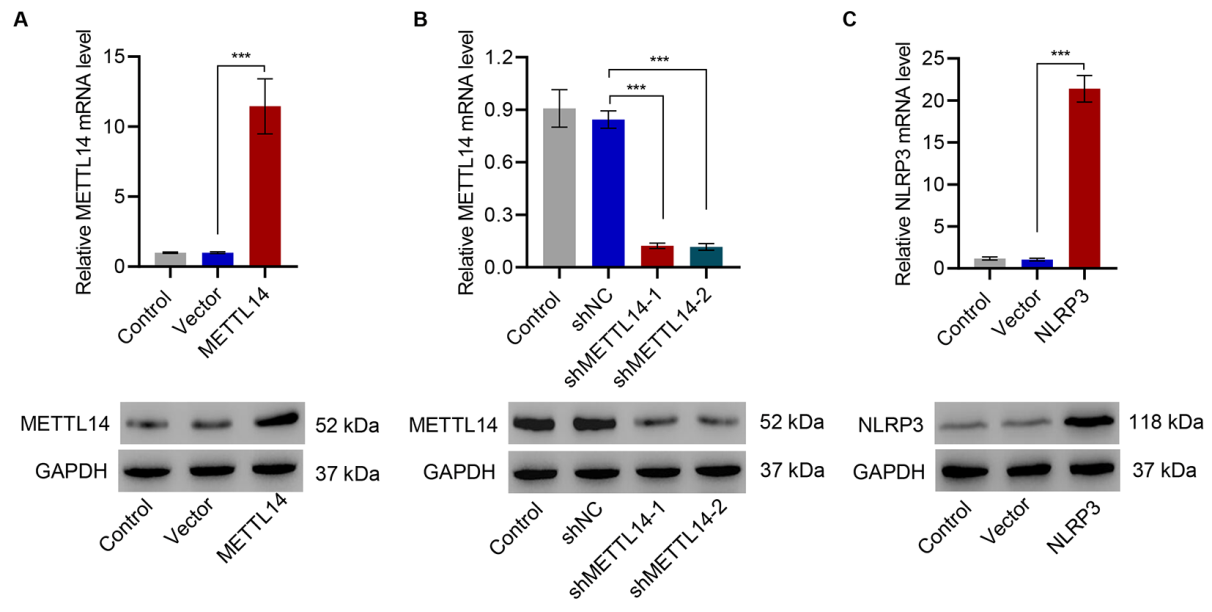


**Figure S3. Expression of METTL14 and NLRP3 in HNPC.** (A-C) Relative expression of METTL14 and NLRP3 in HNPC transduced with METTL14 shRNA vector, METTL14 overexpression vector, or NLRP3 overexpression vector. ****P*<0.001.


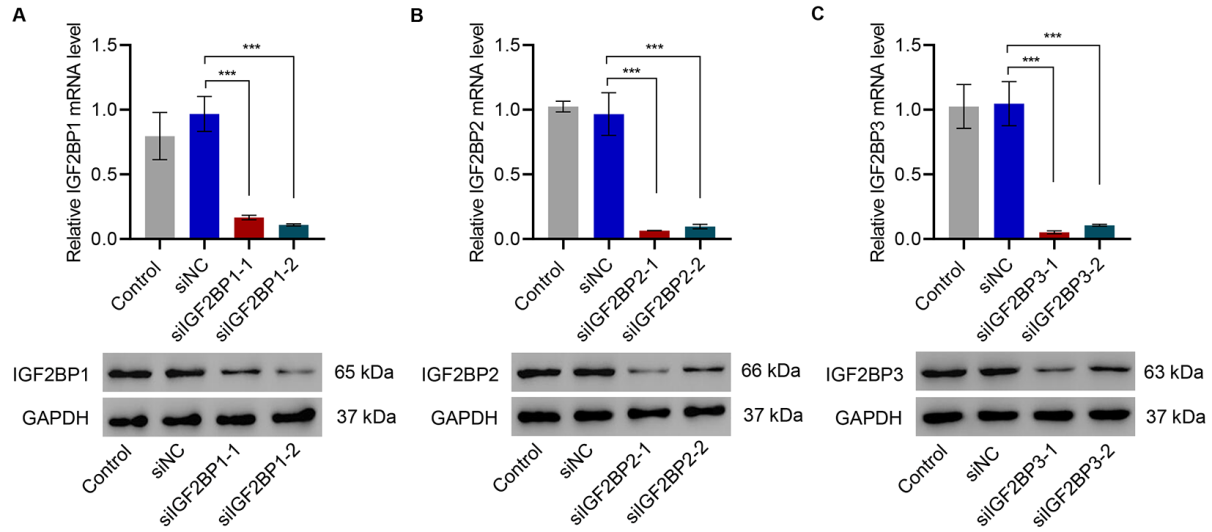


**Figure S4.** **Expression of IGF2BP1, IGF2BP2 and IGF2BP3 in HNPC.** (A-C) Relative expression of IGF2BP1, IGF2BP2, and IGF2BP3 in HNPC transfected with IGF2BP1, IGF2BP2, or IGF2BP3 siRNA. ****P*<0.001.


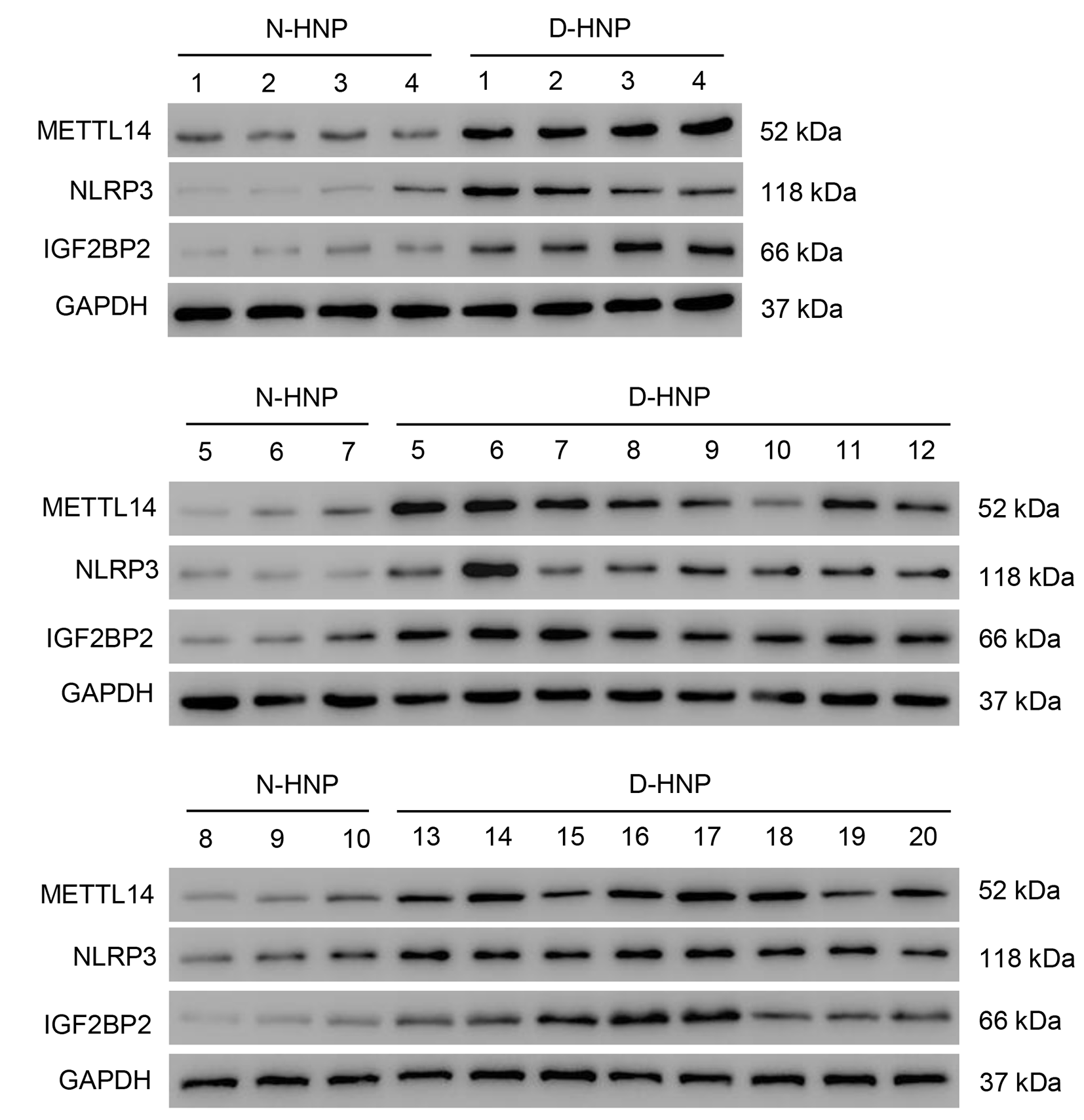


**Figure S5. Expression of METTL14, NLRP3, and IGF2BP2 in HNPC.** Western blot analysis of METTL14, NLRP3, and IGF2BP2 in N-HNP tissues (n = 10) and D-HNP tissues (n = 20).
